# Fluid Resuscitation and Inotropic Support in Patients With Septic Shock Treated in Pediatric Emergency Department: An Open-Label Trial

**DOI:** 10.7759/cureus.30029

**Published:** 2022-10-07

**Authors:** Ricardo Iramain, Jorge Ortiz, Alfredo Jara, Norma Bogado, Rocío Morinigo, Laura Cardozo, Niranjan Kissoon

**Affiliations:** 1 Emergency Department, Hospital de Clinicas, Asunción, PRY; 2 Emergency Department, Hospital de Clínicas, Asunción, PRY; 3 Pediatrics and Emergency Medicine Department, University of British Columbia, Vancouver, CAN

**Keywords:** hypotension, shock, cardiotonic agents, fluid therapy, sepsis

## Abstract

Introduction

Fluid resuscitation and inotropic support are essential interventions to improve cardiovascular function in patients with septic shock. However, the optimal volume of fluids and the timing of inotropic support to achieve the resolution of shock are controversial. They may depend on the availability of critical care support services.

Aims

To compare early versus the delayed start of epinephrine administration after fluids bolus in children with septic shock.

Methods

We conducted an open-label randomized trial in which patients under 18 years of age diagnosed with septic shock and arterial hypotension were treated in two Pediatric Emergency Departments in Paraguay (Hospital de Clinicas of Universidad Nacional de Asunción and Instituto Privado del Niño) between 2015 and 2020. Septic shock was defined according to the American College of Critical Care Medicine (ACCM) guidelines. All patients received antibiotics and 40 ml/kg of fluids (two boluses of 20ml/kg if there were no signs of fluid overload) during the first hour. They were then divided into two groups: Group 1 received epinephrine infusion and maintenance fluids. Group 2 received an additional 20 ml/kg of fluids and then was started on epinephrine infusion.

Results

Of 229 patients screened, 63 patients were included in the study. The mean age was 2.8±3.5 years. A total of 52% were female. Group 1 comprised 33 patients, and group 2 comprised a total of 30. Significant differences were found between group 1 and group 2 in the following: mortality (10% vs. 33%, p: 0.026, RR: 3.1, CI: 95%: 1-10), need for mechanical ventilation (10% vs. 41%, p: 0.006, RR: 4, CI: 95%: 1.3-12), and altered vascular hypoperfusion after one hour of interventions (7% vs. 59%, p<0,001, RR: 8.2, CI: 95%: 2-32).

Conclusions

Early administration of epinephrine infusion after initial fluid therapy was associated with better clinical outcomes than delayed administration.

## Introduction

Sepsis continues to be one of the most frequent causes of morbidity and mortality worldwide, with an estimated 22 cases of children with sepsis per 100,000 people per year and 2202 cases of neonatal sepsis per 100,000 live births, which translates to approximately 1,200,000 children with sepsis each year [1]. Under five years old, children account for 20.3 million cases of sepsis and 2.9 million deaths, which is about 41.5% of cases globally and 26.4% of deaths. Mortality in children with sepsis varies between 4% and 50% depending on the severity, risk factors, and geographic location [2,3].

Sepsis is caused by a response to an infection and is characterized by vital organ dysfunction or failure, and poses a significant threat of death and disability [4]. Septic shock is characterized by the inability of the cardiovascular system to meet cellular metabolic demands. 

Several national and international institutions and scientific societies have developed clinical practice guidelines (CPG) for managing sepsis in children [5,6]. These guidelines emphasize early recognition and early time-sensitive treatments guided by clinical goals. While studies demonstrated improvement in outcomes following its implementation, concerns abound regarding the amount of fluids infused and the timing of inotropic support for those presenting with septic shock [7-9]. 

The major issue relates to the availability of critical care services to provide respiratory support for children in whom overzealous fluid administration may lead to respiratory insufficiency or heart failure with fluid overload. Indeed, the most recent guideline of the Surviving Sepsis Campaign [6] recommends a more cautious approach to fluids and earlier inotropic support in contexts of lower resources countries. Thus, a consensus is evolving for a more cautious approach, especially in resource-poor settings, as seen in many places in Latin America, Sub-Saharan Africa, South Asia, and even in other areas where intensive care support is not readily available [10]. The American College of Critical Care Medicine (ACCM) recommends administering fluid boluses at 20 mL/kg and starting inotropes after 60 mL/kg [5].

The aim of this study is to evaluate short-term clinical outcomes of 40 ml/kg versus 60 ml/kg of fluids before epinephrine administration in similar doses in children with septic shock.

## Materials and methods

We conducted an open-label trial in which patients under 18 years of age were treated in two Pediatric Emergency Departments in Paraguay between 2015 and 2020, with a diagnosis of septic shock and arterial hypotension. The first Pediatric Emergency Department is a University Hospital (Hospital de Clínicas of Universidad Nacional de Asunción) with approximately 50000 Pediatric Emergency Department visits per year. The other is a private hospital (Instituto Privado del Niño) with about 38000 Pediatric Emergency Department visits per year. All children who met our case definition of septic shock (SS) were enrolled. The Ethics Committee of Children's Private Institute reviewed and approved the protocol. Informed consent for participation in the study was obtained from the parents before enrolment.

Septic shock was defined by the American College of Critical Care Medicine clinical guidelines for hemodynamic support of neonates and children with septic shock and the Surviving Sepsis Campaign: International Guidelines for Management of Sepsis and Septic Shock [5,6] as severe infection leading to cardiovascular dysfunction (including hypotension, need for treatment with a vasoactive medication, or impaired perfusion). Septic shock was diagnosed by clinical signs, which include hypothermia or hyperthermia, altered mental status, and peripheral vasodilation with flash capillary refill (warm shock) or vasoconstriction with capillary refill greater than two seconds (cold sho

Inclusion criteria

Patients who had a diagnosis of septic shock hypotension upon admission to the Pediatric Emergency Department were included.

Exclusion criteria

Patients who have been admitted with cardiac arrest, those who received fluid therapy or antibiotic therapy in another center, and those children whose parents did not sign the informed consent were excluded.

Every patient was triaged on arrival at the hospital by a nurse with experience identifying sepsis using the Systemic Inflammatory Response Syndrome (SIRS) [11] criteria. They applied these criteria to patients with a history of fever and/or risk factors. Those who were identified as with suspected sepsis were immediately evaluated by a pediatrician and/or an emergency pediatrician to determine if they fulfilled the criteria using a case form to address their observation. If they were identified as such, they were admitted to the ED shock room, and a pediatrician and/or emergency pediatricians started treatment immediately.

At the admission, demographic and clinical data were collected in a standardized data collection sheet made for the study. Antibiotics were given within the first hour of treatment in all cases. Laboratory tests were performed before starting the treatment (baseline pH, bicarbonate, and blood glucose were done on-site, and procalcitonin, blood culture, and blood lactate level were sent to the laboratory) and then were performed after one hour of treatment.

Hypoperfusion was defined as the presence of decreased or very wide peripheral pulses, slowed capillary refill ≥ seconds or very fast, capillary flash, cold, mottled, or hot and vasodilated extremities, alterations in sensory awareness-somnolence, confusion, or lethargy [5].

Heart rate and blood pressure were measured with a multiparametric monitor, a pediatrician or an emergency pediatrician assessed signs of peripheral vascular perfusion, and urine outcome was measured within the first hour of treatment.

Improved criteria

After each fluid bolus of 20 ml/kg of normal saline in 10-20 minutes, every patient was evaluated for clinical improvement and signs of fluid overload. Normalization of heart rate, respiration rate, blood pressure, and distal pulses; improvement of capillary refill, improved sensorium, and improved urine outcome of >1 ml/kg were considered signs of improvement. Patients who showed minimal or no signs of improvement and in whom there were no signs of fluid overload (enlargement of the liver or rales on chest auscultation) received a second bolus of fluids. After 40 ml/kg (two boluses), children who did not improve were then designed with alternate numbers allocated in a 1.1:1 ratio to group 1 or group 2 (receiving early inotropes vs. standard fluid management) into two groups. Group 1 was started with epinephrine infusion, and group 2 received an additional bolus of 20 ml/kg before starting the epinephrine infusion according to ACCM guidelines. The initial dose of epinephrine was 0.1 μg/kg/min for both groups and was titrated according to cardiovascular response to 0.15 μg/kg/min. Patients who did not improve and continued to be hypoperfused without signs of fluid overload were given extra boluses as needed. 

When patients improved after resuscitation, they continued in the Resuscitation and Stabilization Unit (RSU) for an adequate period, around 12-24 h. Then they were transferred to the common ward for follow-up care. If the patients did not improve, they were transferred to the Pediatric Intensive Care Unity (PICU). 

Outcomes

The primary outcome was a shock resolution using the improved criteria in the first hour of treatment. The secondary outcomes were mortality in the ED, the need for additional fluids and inotropic support, mechanical ventilation, and altered vascular perfusion (defined as persistence of altered capillary refill, weak distal pulse, and cold distal limbs) after one hour of intervention.

The study was carried out following the recommendations established in the Declaration of Helsinki involving human beings.

Statistical analysis

The analysis was performed using Epi Info 7.2.4.0. Nominal variables were presented as frequencies and percentages, continuous variables with a normal distribution were presented as means and SD, and those that did not present a normal distribution were presented as median and interquartile range. The comparison of means was performed using Student's t-test or Mann-Whitney U test as appropriate, and the comparison of nominal variables was performed using the Chi-squared or Fisher's exact test. In all cases, two-tailed tests were performed, and an alpha of 5% and a significant p less than 0.05 were considered. The relative risks were calculated with a 95% CI.

## Results

**Figure 1:**
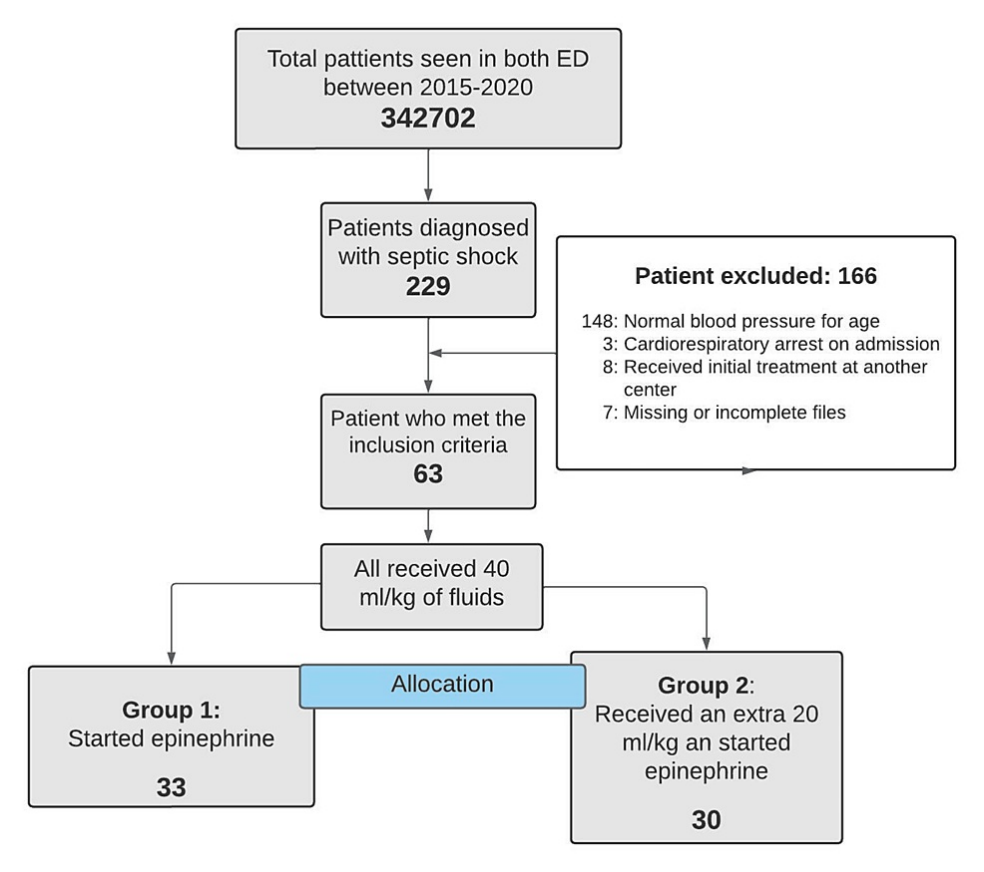
Flowchart of patients included in the study.

In the study period, a total of 229 patients with a diagnosis of septic shock were admitted to both services (Figure 1).

No significant differences were found in clinical characteristics such as age, sex, or comorbidity between the two groups and the baseline laboratory test performed upon admission. Table 1 shows the characteristics of the patients by group.

**Table 1:** Demographic characteristics and baseline laboratory results of patients with septic shock and hypotension treated in two pediatric emergency departments between 2015 and 2020 (n: 63). a: median, b: interquartile range. Group 1: start of epinephrine after 40 ml/kg of fluids, Group 2: start of epinephrine after 60 ml/kg of fluids. Not all blood cultures showed positive.

Baseline characteristics	Group 1 : 40 ml/kg n:33 (%)	Group 2: 60 ml/kg n:30 (%)	P-value
Age (years)	1^a^ (0.4-4)^b^	0.9^a ^(0.2-3)^b^	0.8
Male Sex	19 (57.6)	11 (36.7)	0.097
Comorbidity	11 (18.2)	9 (20)	0.854
Oncological	5 (15.2)	3 (10)	0.54
Cerebral palsy	3 (9)	2 (6.6)	
Diabetes	1 (3)	3 (10)	
Chronic kidney disease	2 (6)	1 (3,3)	
Severe malnutrition	0	2 (6.6)	0.13
Severe Anemia	3 (9)	4 (13.3)	0.5
Site of infection			
Acute gastroenteritis	8 (24.2)	11 (36.7)	0.283
CNS	5 (15.2)	2 (6,7)	0.195
Urinary infection	10 (30.3)	8 (26.7)	0.425
Respiratory	13 (39.4)	13 (43.3)	0.751
Blood cultures			
Gram (+)	7 (21.2)	9 (30)	0.719
Gram (-)	13 (39.4)	10 (33.3)	
Negative culture	13 (39.4)	11 (36.7)	
Epinephrine initiation (time from the diagnosis)			
30 min	20 (66.7)	22 (60.6)	0.284
40 min	13 (43.3)	8 (39.4)	
Laboratory results at baseline			
pH	7.2 ± 0.7	7.03 ± 0.6	0.7
Bicarbonate (mEq/L)	9.8 ± 1.1	9.7 ± 1.0	0.59
Lactic acid (mmol/L)	2.4 ± 0.2	2.3 ± 0.3	0.3
Procalcitonin (ng/mL)	2.9 ± 0.7	2.7 ± 0.9	0.3
Blood glucose (mg/dL)	100 ± 31	106 ± 25	0.4

After the initial 40 ml/kg saline fluids were performed, a significant difference was observed between the mean values of pH, bicarbonate, and lactic acid between the two groups. The laboratory results are summarized in Table 2.

**Table 2:** Comparison by group of mean values of laboratory results after initial treatment in children with septic shock (n: 63).

Laboratory results	Group 1 n:33 Mean ± DE	Group 2 n:30 Mean ± DE	P-value
pH	7.2 ± 0.1	7.1 ± 0.1	<0.001
Bicarbonate (mEq/L)	15.4 ± 3.7	10.8 ± 1.1	<0.001
Lactic acid (mmol/L)	2.1 ± 0.4	2.4 ± 0.4	0.016

More children in group 2 versus group 1 received mechanical ventilation (group 1: 10% vs. group 2: 41%, p:0.006, RR: 4, 95% CI: 1.3-12). A significantly higher mortality rate was also found in group 2 (group 2: 33% vs. group 1: 10% (p: 0.04), RR: 3.1; 95% CI: 1-10). In addition, decreased peripheral vascular perfusion one hour after the start of the interventions was higher in group 2 versus group 1 (group 2: 59% vs. group 1: 7% (p <0.001, RR: 8.2 95% CI: 2-32) and therefore had to be given more boluses of fluids.

## Discussion

We found higher mortality and fewer instances of shock resolution between patients who received earlier epinephrine infusion after 60 ml/kg fluid bolus as compared with those who received 40 ml/kg. There was a greater need for mechanical ventilation and additional fluid boluses to improve altered vascular perfusion in group 2.

Recently several international guidelines have been developed for the management of septic shock. All emphasize the importance of prompt initiation of vascular access, IV fluids, and inotropes [12-14]. However, the time of inotropic initiation after fluid resuscitation is not standardized and depends on resource availability. It has been suggested that if the fluid dose between 40 and 60 ml/kg is reached without improvement, the infusion of vasoactive drugs should be started between 30 and 60 minutes before the beginning of the treatment of septic shock [9]. To our knowledge, few published studies have evaluated the clinical impact of early inotropic administration after fluid infusion at 40 ml/kg compared to 60 ml/kg. Santhanam I et al. [15] compare the impact of 40 mL/kg of fluid over 15 minutes followed by dopamine versus 20 mL/kg over 20 minutes up to a maximum of 60 mL/kg over one hour followed by dopamine in septic shock. However, they did not find any difference in the overall mortality, rapidity of shock resolution, or incidence of complications between the groups. We have compared epinephrine as an inotropic drug without considering the times.

Upadhyay M et al. [16] found that the most important cause of septic shock was pneumonia, like our finding. Sankar J et al. [17] compared the effect of the administration of 40-60 mL/kg of fluids as a bolus in aliquots of 20 mL/kg each over 15-20 minutes with that over 5-10 minutes each on the composite outcome of need for mechanical ventilation and/or impaired oxygenation-increase in oxygenation index by five from baseline in the initial 6 and 24 hours in children with septic shock. They found that children receiving fluid boluses over 5-10 minutes each had a higher risk of intubation than those receiving boluses over 15-20 minutes each. Based on concerns of bolus overload, we administered fluids within 30-40 minutes before starting inotropes, not finding volume overload.

The mean value of lactic acid was significantly higher in group 2, reflecting this group's more critical clinical situation. This finding agrees with other studies that support that elevated lactate is a predictor of mortality in an ED [18]. The ACCM [5] guidelines have suggested that lactate cannot be used as a marker of the severity of shock but could be useful to judge an improvement in perfusion as a response to fluid resuscitation. Furthermore, Jat KR et al. [19] have shown that high levels of lactic acid are a predictor of poor tissue perfusion and poor outcomes in patients with septic shock.

The present study found that patients who received earlier epinephrine had a significantly greater probability of survival; those of group 2 were three times more likely to die (10% vs. 33%). The persistence of altered peripheral vascular perfusion one hour after the start of the interventions in group 2 and the poorer outcomes indicate suboptimal resuscitation. This is supported by Han YY et al. [20], who reported a two-fold increase in mortality for each hour in which the shock persisted and stated that the use of time dependent on the guidelines that point to the early use of inotropic could reduce mortality. Ninis N et al. [21] described an association between inotropic delay and a two-fold increase in mortality in septic shock due to meningococcemia. Carcillo JA et al. [22] demonstrated that the early reversal of shock is related to applying the pediatric advanced life support (PALS) guidelines and that each hour that passes without implementing it increases mortality by 40%. None of these studies, however, referred to comparing the boluses and timing of inotropes. Despite recent advances in the management of sepsis, mortality remains unacceptably high, with rates ranging between 10% and 51% [21-22]. Our finding of a 33% mortality rate is similar to Tan B et al. [3], who reported that septic shock and sepsis have a 32% fatality rate in low- to middle-income countries such as Paraguay; these findings coincide with other authors [23,24]. Among the limitations of our study are the small number of participants and the absence of follow-up of the patients to determine any long-term differences. In addition, we were unable to use PICU admission as an outcome due to the limitations of PICU unit availability in our country. However, it should also be considered that both participating centers come from a country with low incomes and limited ICU access, so the results can be extrapolated to similar situations. According to our knowledge, this is the first study demonstrating the efficacy of starting the inotropic support after 40 ml/kg compared to 60 ml/kg as advocated by some international guidelines in the management of pediatric septic shock in the first hour. 

## Conclusions

Our study found that early administration of epinephrine infusion after initial fluid therapy in children with septic shock was associated with better clinical outcomes than delayed administration. These findings require further studies with a larger population to establish the appropriate time to start inotropics in children with septic shock.
